# A direct regulatory link between microRNA-137 and *SHANK2*: implications for neuropsychiatric disorders

**DOI:** 10.1186/s11689-018-9233-1

**Published:** 2018-04-17

**Authors:** Ana de Sena Cortabitarte, Simone Berkel, Flavia-Bianca Cristian, Christine Fischer, Gudrun A. Rappold

**Affiliations:** 1 Institute of Human Genetics, Ruprecht-Karls-University, Heidelberg, Germany; 2 Interdisciplinary Center for Neurosciences (IZN), Ruprecht-Karls-University, Heidelberg, Germany; 3 Department of Human Molecular Genetics, Institute of Human Genetics, Im Neuenheimer Feld 366, 69120 Heidelberg, Germany

**Keywords:** *SHANK2*, microRNA, miR-137, Schizophrenia, Autism, Intellectual disability

## Abstract

**Background:**

Mutations in the *SHANK* genes, which encode postsynaptic scaffolding proteins, have been linked to a spectrum of neurodevelopmental disorders. The *SHANK* genes and the schizophrenia-associated microRNA-137 show convergence on several levels, as they are both expressed at the synapse, influence neuronal development, and have a strong link to neurodevelopmental and neuropsychiatric disorders like intellectual disability, autism, and schizophrenia. This compiled evidence raised the question if the SHANKs might be targets of miR-137.

**Methods:**

In silico analysis revealed a putative binding site for microRNA-137 (miR-137) in the *SHANK2* 3′UTR, while this was not the case for *SHANK1* and *SHANK3*. Luciferase reporter assays were performed by overexpressing wild type and mutated SHANK2-3′UTR and miR-137 in human neuroblastoma cells and mouse primary hippocampal neurons. miR-137 was also overexpressed or inhibited in hippocampal neurons, and *Shank2* expression was analyzed by quantitative real-time PCR and Western blot. Additionally, expression levels of experimentally validated miR-137 target genes were analyzed in the dorsolateral prefrontal cortex (DLPFC) of schizophrenia and control individuals using the RNA-Seq data from the CommonMind Consortium.

**Results:**

miR-137 directly targets the 3′UTR of *SHANK2* in a site-specific manner. Overexpression of miR-137 in mouse primary hippocampal neurons significantly lowered endogenous Shank2 protein levels without detectable influence on mRNA levels. Conversely, miR-137 inhibition increased Shank2 protein expression, indicating that miR-137 regulates *SHANK2* expression by repressing protein translation rather than inducing mRNA degradation.

To find out if the miR-137 signaling network is altered in schizophrenia, we compared miR-137 precursor and miR-137 target gene expression in the DLPFC of schizophrenia and control individuals using the CommonMind Consortium RNA sequencing data. Differential expression of 23% (16/69) of known miR-137 target genes was detected in the DLPFC of schizophrenia individuals compared with controls. We propose that in further targets (e.g., *SHANK2*, as described in this paper) which are not regulated on RNA level, effects may only be detectable on protein level.

**Conclusion:**

Our study provides evidence that a direct regulatory link exists between miR-137 and *SHANK2* and supports the finding that miR-137 signaling might be altered in schizophrenia.

**Electronic supplementary material:**

The online version of this article (10.1186/s11689-018-9233-1) contains supplementary material, which is available to authorized users.

## Background

microRNAs are small non-coding, single-stranded RNA molecules that regulate gene expression by binding to the 3′UTRs of their target mRNAs through base pairing of their 6–8-nucleotide long seed region. They either initiate mRNA degradation or inhibit protein translation [1, 2]. Regulation of gene expression in the nervous system is highly complex and is fine-tuned by microRNAs [3]. In neurons, mRNAs and microRNAs are compartmentalized to specific subcellular regions, like the synaptodendritic compartment, to regulate local protein synthesis in response to neuronal stimuli [4].

microRNA-137 (miR-137) is enriched in the human and mouse brain [5], especially in cortical regions and in the hippocampus [6]. It plays a role in cell proliferation and differentiation and is present at the synapse [6, 7]. Furthermore, it regulates neuronal maturation, dendritic morphogenesis, and spine development [8]. Homozygous knockout of *Mir137* is embryonically lethal in mice, indicating that embryonic development is dependent on at least one functional allele [9]. This emphasizes the importance of miR-137 in early developmental processes.

Genetic alterations of *MIR137* have been associated with neurodevelopmental disorders. The rare chromosomal microdeletion 1p21.3 encompassing *MIR137* and *DPYD* was identified in individuals with intellectual disability (ID), comorbid with autism spectrum disorder (ASD), and obesity [6, 10, 11]. In addition, 19/65 (29%) highest ranked ASD risk genes in the Simons Foundation Autism Research Initiative gene database [12, 13] (URL: https://sfari.org/resources/sfari-gene#refs, accessed May 2017) are predicted or confirmed miR-137 targets (Additional file 1: Table S1). miR-137 target genes are enriched among ASD risk genes (29%, CI 18.6–41.8%) compared with the random frequency (2.9%, predicted in the miRDB database [14]; CI 2.7–3.2%, 525/17860) (*P* ≤ 0.000001, Fisher’s exact test, two-sided). Furthermore, the *MIR137* gene has been reported as a schizophrenia (SCZ) susceptibility locus because the common SNP rs1625579, located in an intron of *MIR137,* was associated with SCZ in several studies [15–18]. Putative miR-137 targets are enriched in SCZ risk loci [18], suggesting that convergent pathways connected by this microRNA contribute to SCZ etiology. A meta-analysis of SNP data across five different neuropsychiatric disorders identified a shared risk locus association with SCZ and ASD [19]. Taken together, these findings indicate that deregulation of miR-137 is the key to various psychiatric and neurodevelopmental disorders.

SHANK proteins (SHANK1, SHANK2, and SHANK3) are postsynaptic scaffolding proteins with an important role in the formation, maintenance, and function of synapses in glutamatergic neurons of the brain [20]. *SHANK* genes converge with miR-137 on several levels: (i) both are expressed at the synapse, (ii) both influence dendrite and spine formation in glutamatergic neurons [8, 21], and (iii) both have a strong link to neurodevelopmental and neuropsychiatric disorders like ID, ASD, and SCZ [18, 22, 23]. This suggests that *SHANK* genes might be targets of miR-137.

## Methods

### Luciferase assays

To validate a predicted miR-137 binding site in *SHANK2*, luciferase reporter assays were carried out in human SH-SY5Y cells and mouse primary hippocampal neurons. The wild type *SHANK2*-3′UTR was cloned into the psiCHECK™-2 vector (Promega, Mannheim, Germany) after PCR amplification on human genomic DNA from a healthy individual. PCR primers introduced the restriction sites (XhoI) which were used to introduce the PCR product into the psiCHECK™-2 vector via Gibson® Assembly (New England BioLabs, Frankfurt, Germany). Mutagenesis of the miR-137 binding site was performed on the wild type construct by PCR with the original cloning primers and mutagenesis primers, assembled using the SLiCE cloning system [24]. The sequence of wild type and mutated SHANK2-3′UTR was validated by Sanger sequencing. Primer sequences are given in Additional file 1: Table S2.

The SH-SY5Y cells were seeded in antibiotic-free medium and transfected after 24 h with 200 ng of empty psiCHECK™-2 vector and wild type or mutated *SHANK2* 3′UTR constructs with 12 nM miR-137 or control mimics (mirVana® miRNA hsa-miR-137 mimic MIMAT0000429 and Pre-miR™ miRNA Precursor Molecules Negative Control #2, AM17111, Thermo Fisher Scientific, Darmstadt, Germany) in technical triplicates using Lipofectamine®2000 (Thermo Fisher Scientific, Darmstadt, Germany). miRNA mimics are small, double-stranded RNA molecules which mimic endogenous, mature microRNA molecules when introduced into the cells. Luciferase activity was measured 48 h posttransfection using the dual luciferase reporter assay system (Promega) and the luminometer Centro LB960 (Berthold Technologies, Bad Wildbad, Germany). Renilla luciferase activity was normalized against firefly luciferase activity, and the background was subtracted by normalization to the empty psiCHECK™-2 vector activity.

Primary hippocampal neurons were transfected on day in vitro (DIV) 5 using the same experimental setup as in the SH-SY5Y cells for the mimic experiments with 25 nM mimics. The amount of miR-137 mimics had to be optimized for each cell type separately. For the inhibitor experiments, the primary mouse hippocampal neurons were treated with 25 nM miR-137 or power inhibitor control (miRCURY LNA™ Power microRNA hsa-miR-137 4101446-111 or negative control B 199007-111 inhibitor 5′-Fluorescein-labeled, Exiqon, Vedbaek, Denmark) added drop-wise to the cells on DIV5. The miR-137 power inhibitor sequesters both the human hsa-miR-137 and mouse mmu-miR-137, thereby preventing it from binding to its targets*.* The control inhibitor does not influence miR-137 and was used to control for unspecific effects of the treatment. Experimental conditions were identical between both groups regarding cell number and cell types (Additional file 1: Figure S1). The cells were transfected with the luciferase reporter constructs with Lipofectamine®2000 on DIV6. Luciferase activity was measured 48 h posttransfection as described above.

### Cell culture

The SH-SY5Y cells were obtained from the DSMZ (Leibniz Institute German collection of Microorganisms and Cell Cultures, Braunschweig, Germany, ACC209). The cells were kept in supplemented Dulbecco’s Modified Eagle’s Medium (DMEM) (15% fetal bovine serum (FBS), 1% penicillin/streptomycin (P/S), 1% non-essential amino acids). Hippocampal cultures were prepared from gestating CD-1® IGS mice (Charles River, Sulzfeld, Germany) at embryonic stage 15.5. The cells were seeded in plating media (DMEM + 10% FBS, 0.25% P/S, 2 mM L-glutamine) on poly-L-ornithine (Sigma Aldrich, Schnelldorf, Germany) coating. On DIV 1, a complete medium change to supplemented Neurobasal medium (Gibco® Neurobasal® media + 2% Gibco®B-27® supplement, 0.25% P/S, 0.5 mM L-glutamine) was performed. On DIV3, a half medium change was carried out with 5 μM cytosine β-D-arabinofuranoside in supplemented Neurobasal media, except for the cells that were harvested on DIV3. From DIV5 until the final culture day, a half medium change was performed with supplemented Neurobasal media every other day, except for the cells that were transfected or harvested.

### Nucleofection of mouse hippocampal cells

Hippocampal cultures were transfected with the optimized hsa-miR-137 mimic concentration of 300 nM and negative miRNA control on DIV5 using the AD1 4D-Nucleofector™ Y Unit system (Lonza, Basel, Switzerland) according to manufacturer’s instructions. Cell types and cell numbers showed no difference after nucleofection between the two conditions (Additional file 1: Figure S1).

### RT-qPCR

To determine the effect of miR-137 on endogenous *Shank2* expression real-time quantitative PCR (RT-qPCR) was performed. For the quantification of *Shank2* expression, RNA was isolated from mouse primary hippocampal neurons (DIV8) 72 h after transfection with using the Quick-RNA™ MicroPrep Kit (Zymo Research, Freiburg, Germany) and was transcribed with the SuperScript® *VILO*™ cDNA Synthesis Kit (Thermo Fisher Scientific, Darmstadt, Germany). RT-qPCR was performed to quantify *Shank2* expression normalized against two reference genes (*Gapdh* and *Hprt1*). Primer sequences are provided in Additional file 1: Table S2.

The RNAqueous®-Micro Total RNA Isolation Kit (Thermo Fisher Scientific) was used for total RNA isolation. Ten nanograms of total RNA were transcribed using the miRCURY LNA™ microRNA PCR Universal cDNA synthesis Kit (Exiqon) with a spike-in loading control according to manufacturer’s instructions. RT-qPCR was performed with miRCURY LNA™ microRNA PCR ExiLENT SYBR® Green PCR sets for hsa-miR-137, U6, and spike-in (Exiqon), measuring the endogenous miR-137 expression profile in primary hippocampal neurons over a time period of 11 DIV. miR-137 expression levels were analyzed with the same method using 21 different human tissue RNA samples (specified in Additional file 1: Table S3).

### Western blot analysis

To determine the effect of miR-137 on Shank2 protein expression, cellular protein was isolated using RIPA buffer from cell cultures on DIV10, and the lysates were run on Novex™WedgeWell™4–12% Tris-Glycine Gels (Thermo Fisher Scientific) and then blotted onto a PVDF membrane (Immobilon-FL, Millipore, Billerica, Massachusetts, USA) as recommended by the manufacturer. Primary antibodies anti-beta-III-tubulin (mouse, G7121 Promega) and anti-Shank2 (rabbit, ABIN656710 antibodies-online) were used followed by the secondary IRDye 680 donkey anti-mouse and IRDye 800CW donkey anti-rabbit antibodies (1706515 and 1706516, respectively, Li-COR). The membrane was scanned with the Odyssey® Infrared Imaging System (Li-COR) and quantified using ImageJ software^39^. Shank2 was normalized to beta-III-tubulin. All experiments were performed sequentially over several weeks. Hippocampal neurons were isolated and differentiated at different time points.

### CommmonMind data

The tissue used for RNA sequencing was obtained from the following institutes: Mount Sinai School of Medicine, University of Pennsylvania, University of Pittsburgh, National Institute of Mental Health, F. Hoffman-La Roche Ltd., Takeda Pharmaceuticals Company Limited, Sage Bionetworks, Duke University, and University of North Carolina. Generation and analysis of the RNA sequencing data has been previously described in detail by the CommonMind Consortium [25]. To analyze the expression levels of the validated miR-137 target genes and miR-137 precursor, we selected the average expression levels, the log_2_-fold change and the *P* values for the respective genes published on https://synapse.org in the “Results Explorer” on the “CommonMind Consortium Knowledge Portal” (data accessed June 2017). A weighted linear regression analysis had been performed for each gene considering covariates that influence expression levels. For each gene, the SCZ disease status coefficient had been statistically tested for being nonzero, implying an estimated effect for SCZ, above and beyond any other effect from covariates. This test produced a *t* statistic and a corresponding *P* value. Details about the statistical analysis are provided in the CommonMind Consortium paper [25].

### Selection of experimentally validated miR-137 targets and network analysis

Validated targets of miR-137 were collected from miRTarBase (http://mirtarbase.mbc.nctu.edu.tw/, accessed January 2017) and PubMed (https://www.ncbi.nlm.nih.gov/pubmed/, accessed January 2017) database searches. miRTarBase targets were included if they were categorized as “strong evidence,” i.e., confirmed by experimental methods of validation including luciferase reporter assay, RT-qPCR, and/or Western blot analysis. Targets from the PubMed database search were considered as validated if a direct link was established by luciferase reporter assay, RT-qPCR, and/or Western blot analysis. Duplicates between databases were removed. A total of 71 validated targets were obtained; most of them have been investigated in cancer cells, whereas only 12 targets have been validated in neurons (details are shown in Additional file 1: Table S4). RNA sequencing data for the miR-137 target genes *HCRT*, *SLC6A3*, and *SNAI1* was not available in the CommonMind dataset. The target gene network was analyzed through the use of QIAGEN’s Ingenuity®Pathway Analysis (IPA®, QIAGEN Redwood City, www.qiagen.com/ingenuity).

### Statistical analysis

Statistical analyses were performed using the SPSS software (IBM Corp.; IBM SPSS Statistics for Windows, Version 22.0.; Armonk, NY, USA). Two-way ANOVAs were performed considering the influence of the experiment and the respective condition. The data is shown as mean ± SEM. The validated miR-137 target genes (including *SHANK2*) were ranked according to their point-wise *P* values from the CommonMind analysis, and the Benjamini-Hochberg method was used to correct for multiple testing with a false discovery rate of 10% (Additional file 1: Table S5). A two-sided *Χ*^2^ test with Yates correction was used to compare the expression of validated target genes of miR-137 and five control microRNAs. The differentially expressed targets of the five control microRNAs were pooled together. Genes that were targets of more than one control microRNA were only counted once. Eight target genes that were not differentially expressed in SCZ and control individuals overlapped between miR137 and the five controls and were excluded from analysis.

## Results

### miR-137 directly targets the *SHANK2* 3′UTR

In silico analysis (TargetScanHuman Release 7.1 [26]) of all three *SHANK* genes identified a single, highly conserved binding site for miR-137 (MIMAT0000429) in the 3′UTR of *SHANK2* (ENST00000449833.2, Additional file 1: Figure S2A), suggesting that this microRNA has the potential to bind *SHANK2* mRNA. The analysis of miR-137 expression in 21 different human tissue samples revealed high expression in the central nervous system and marginal expression in other tested organs (Additional file 1: Figure S3). miR-137 was highly expressed in the fetal brain, and expression remained strong in the adult hippocampus, thalamus, and striatum. These are brain regions where *SHANK2* is also expressed [27, 28].

**Fig. 1:**
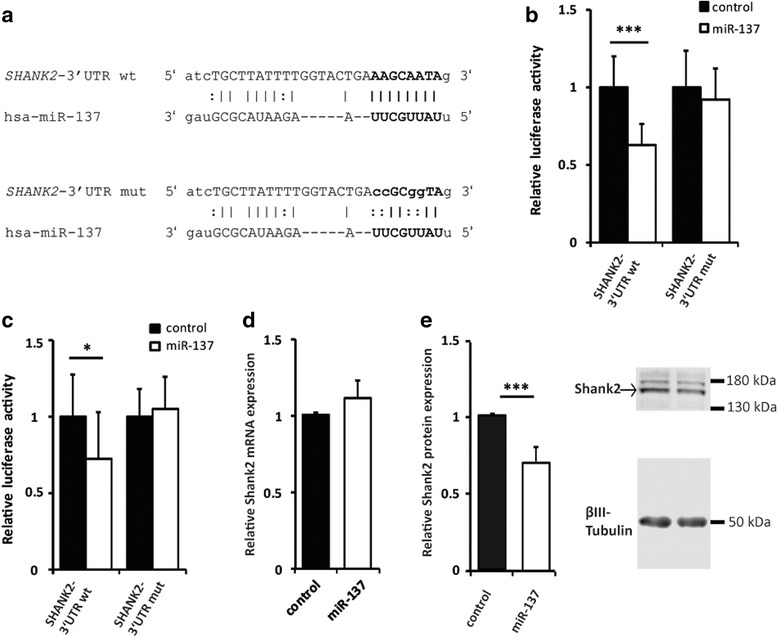
**a** Alignment of miR-137 with the human *SHANK2-*3′UTR (NM_012309) wild type and mutated seed sequence. **b** Luciferase activity using the wild type (wt) and mutated (mut) 3′UTR of *SHANK2* co-transfected with hsa-miR-137 mimic or control miRNA in the SH-SY5Y cells. The data was normalized to the empty psiCHECK™-2 vector, relative to the control miRNA. **c** Luciferase gene activity was measured 48 h after co-transfection of wild type and mutated *SHANK2* 3′UTR with hsa-miR-137 or control miRNA in primary mouse hippocampal cultures (DIV5). **d** Relative *SHANK2* expression levels were measured by RT-qPCR. Primary mouse hippocampal neurons were transfected with either hsa-miR-137 or control miRNA. RNA was harvested 48 h posttransfection (*n* = 3 experiments). **e** Western blot of primary mouse hippocampal neurons 5 days posttransfection with hsa-miR-137 or control miRNA. Cropped pictures indicate Shank2 and βIII-tubulin expression and the full length blots are presented in Additional file 1: Figure S4A. The data was normalized to the control miRNA. Bar plots show mean ± SEM; ***P < 0.001, *P < 0.05 two-way ANOVA; **b**, **c**, and **e** (*n* = 5 experiments)

To validate the predicted miR-137 binding site in *SHANK2*, we carried out luciferase reporter assays. Human neuroblastoma cells (SH-SY5Y) were transfected with a dual luciferase reporter plasmid (psiCHECK™-2) containing either the wild type (wt) *SHANK2* 3′UTR sequence or a miR-137 binding site-mutated 3′UTR (mut) together with either miR-137 or negative control miRNA mimics (Fig. 1a). The SH-SY5Y cells co-transfected with *SHANK2* wild type 3′UTR reporter and miR-137 showed a 38% decrease in luciferase activity compared with the negative control (Fig. 1b; ***P = 5.2 × 10^−9^, two-way ANOVA). In contrast, no difference was observed between miR-137 and negative control miRNA for the mutated 3′UTR. This indicated that miR-137 specifically binds the target site, as mutation of the seed region in the miR-137 binding site eliminated the robust downregulatory effect of miR-137.

Alterations in the microRNA machinery have been described in various cancers [29]; therefore, we further analyzed the regulatory effect of miR-137 on *Shank2* expression in mouse primary hippocampal neurons. These neurons express *Shank2* and *Mir137*, and the miR-137 binding site is conserved in the mouse *Shank2* 3′UTR (Additional file 1: Figure S2B). We co-transfected *SHANK2* wild type 3′UTR reporter and miR-137 and observed a 28% decrease in luciferase activity compared with the negative control (*P = 0.022, two-way ANOVA). In contrast, no effect was seen with the mutated 3′UTR (Fig. 1c). We concluded that miR-137 directly and specifically targets the predicted binding site in the 3′UTR of *SHANK2* in the SH-SY5Y cells and primary hippocampal neurons.

### miR-137 regulates Shank2 protein levels

We overexpressed miR-137 and negative control miRNA mimics in mouse hippocampal neurons to determine the effect on endogenous *Shank2* expression. miR-137 overexpression did not alter endogenous *Shank2* mRNA levels in hippocampal neurons (Fig. 1d). However, Western blotting revealed a 29% reduction of Shank2 protein (***P = 0.00003, two-way ANOVA) after miR-137 overexpression in hippocampal neurons compared with the negative control miRNA mimics (Fig. 1e).

**Fig. 2:**
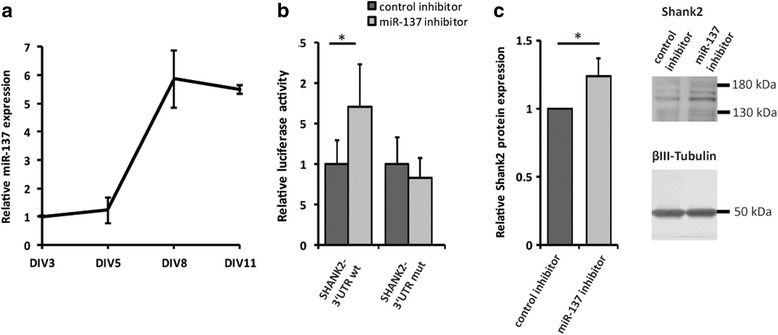
**a** Expression profile of miR-137 in mouse primary hippocampal cultures over 11 days in vitro (DIV). The graph shows data relative to DIV3 (*n* = 3). **b** Luciferase assay in primary mouse hippocampal neurons treated with hsa-miR-137 or control power inhibitors at DIV5. Luciferase reporter constructs were transfected at DIV6, and luciferase activity was measured after 48 h. **c** Western blot with lysate from primary mouse hippocampal neurons treated with hsa-miR-137 or control power inhibitor at DIV5 and protein was harvested after 5 days. Cropped pictures indicate Shank2 and βIII-tubulin expression, and the full length blots are presented in Additional file 1: Figure S4B. Bar plots show mean ± SEM; *P < 0.05 two-way ANOVA; (*n* = 5 experiments)

To determine whether endogenous miR-137 is able to regulate *Shank2* expression, we carried out luciferase assays and Western blot experiments in mouse primary hippocampal neurons after inhibition of miR-137 with a targeted miRCURY LNA™ Power microRNA inhibitor or treatment with a microRNA inhibitor control. First, we analyzed the endogenous miR-137 expression profile in primary hippocampal neurons. Expression levels remained constant until 5 days in vitro (DIV5), increased six-fold between DIV5 and DIV8, and remained high until DIV11 (Fig. 2a). Next, we treated cells with a miR-137 inhibitor or control inhibitor on DIV5, shortly before miR-137 reaches its highest endogenous expression levels. We performed luciferase reporter assays on DIV6 using SHANK2 3′UTR luciferase constructs. miR-137 inhibition increased the relative luciferase activity for the SHANK2 wt reporter construct by 70% (*P = 0.044, two-way ANOVA) compared with the control (Fig. 2b). Endogenous miR-137 was sequestered by the inhibitor; therefore, the regulatory effect on the *SHANK2* 3′UTR was lost and luciferase activity was increased. This was not observed with the mutated *SHANK2* 3′UTR reporter construct. Finally, we analyzed the consequences of endogenous miR-137 inhibition on Shank2 protein levels by Western blot analysis. Inhibition of miR-137 significantly increased endogenous Shank2 protein expression by 24% compared with control inhibitor (*P = 0.016, two-way ANOVA) (Fig. 2c).

### Expression of miR-137 precursor and validated miR-137 target genes in the DLPFC of SCZ and control individuals

Previous studies have pointed to cellular, neurochemical and functional abnormalities in the dorsolateral prefrontal cortex (DLPFC) of SCZ individuals, implicating a role of this particular brain region in the pathophysiology of SCZ [30]. In the DLPFC, *SHANK2* and miR-137 are both expressed [27, 31] and we aimed to identify whether miR-137, *SHANK2*, and other known miR-137 targets are differentially expressed in the DLPFC of SCZ individuals compared to controls. We used a gene expression resource that provides RNA sequencing data in the DLPFC of deceased individuals with SCZ (*n* = 258) and control (*n* = 279) subjects (CommonMind Consortium) [25]. Out of 16,423 genes analyzed, 693 genes were differentially expressed between SCZ and control individuals; 332 were upregulated and 361 were downregulated [25]. The data for miR-137 precursor but not mature miR-137 expression levels were available in this data set. We examined the data for miR-137 precursor expression and found that it did not differ between SCZ and control individuals (MIR137HG, ENSG00000231269, *P* = 0.699). Next, we focused on the miR-137 signaling network and analyzed the expression of 69 known miR-137 target genes (68 validated targets from previously published studies summarized in Additional file 1: Table S4 and *SHANK2*). In total, 16 out of 69 genes were differentially expressed using the Benjamini-Hochberg method to correct for multiple testing with a false discovery rate of 10% (Table 1, Additional file 1: Table S5). This analysis was based on differential expression at the gene level. Four target genes (4/16; *RORA*, *CPLX1*, *TCF4*, *SIRT1*) showed significant expression differences on the whole-transcriptome level [25]. All study-wide significant miR-137 target genes had modest fold changes with a mean of 1.09 and a range of 1.049–1.135 (inverting downregulated expression ratios). The majority of differentially expressed miR-137 target genes (12/16) showed elevated mRNA levels in SCZ individuals compared with controls. Five of the 12 upregulated genes *(TCF4*, *SIRT1*, *XIAP*, *GRIA1*, *ZNF804A*) had already been associated with SCZ. In addition, genes linked to ID (*TCF4*, *CPLX1*, *CDK6*, *KDM5B*), ASD (*RORA*, *KDM5B*), and bipolar disorder (*ZNF804A*) were also among the 16 differentially expressed target genes. Elevated mRNA expression of the SCZ-associated gene *GRIA1* has been described in layer II/III and V pyramidal cells of the DLPFC in SCZ individuals [32].

**Table 1:** Experimentally validated miR-137 target genes (summarized in Additional file 1: Table S4) with a significant expression difference in the DLPFC (dorsolateral prefrontal cortex) between SCZ and control individuals. The log_2_-fold change and *P* values were taken from the analysis published by the CommonMind Consortium (summarized in Additional file 1: Table S5). The log_2_-fold change was calculated by adding 1 to the respective values. The Benjamini-Hochberg method was used to correct for multiple testing with a false discovery rate of 10%. SCZ schizophrenia, ASD autism spectrum disorders, BD bipolar disorder, ID intellectual disability

Gene symbol	Ensemble ID	Log_2_-fold change	*P* value	Association to neurodevelopmental/neuropsychiatric disease	Ref.
*RORA*	ENSG00000069667	1.118	1.21E−05	ASD candidate	[43]
*CPLX1*	ENSG00000168993	0.820	4.60E−04	Wolf Hirschhorn syndrome	[44]
*TCF4*	ENSG00000196628	1.063	4.97E−04	SCZ, Pitt-Hopkins syndrome	[18, 45]
*SIRT1*	ENSG00000096717	1.074	5.59E−04	SCZ	[46]
*ESRRA*	ENSG00000173153	0.888	2.72E−03		
*CDK6*	ENSG00000105810	1.087	3.86E−03	Microcephaly	[47]
*XIAP*	ENSG00000101966	1.060	4.68E−03	SCZ	[48]
*ZNF804A*	ENSG00000170396	1.108	5.29E−03	SCZ, BD	[49]
*MET*	ENSG00000105976	1.132	5.34E−03		
*CTBP1*	ENSG00000159692	0.936	5.77E−03		
*GRIA1*	ENSG00000155511	1.068	8.78E−03	SCZ	[32, 50]
*KLF12*	ENSG00000118922	1.066	1.10E−02		
*MSI1*	ENSG00000135097	0.865	1.23E−02		
*KDM5B*	ENSG00000117139	1.049	1.45E−02	ID, ASD	[51]
*PAQR3*	ENSG00000163291	1.067	1.71E−02		
*TRIM13*	ENSG00000204977	1.050	2.28E−02		

In total, 23% of miR-137 targets were differentially expressed between SCZ and control individuals. We compared the expression of these targets to other microRNA targets. We selected the following five microRNAs that are expressed in the DLPFC but are not differentially expressed in SCZ individuals: let-7a, miR-21-5p, miR-93-5p, miR-451a, and miR-675-5p. Expression data for these microRNA precursors was not available in the CommonMind data set. We analyzed the number of validated target genes that were differentially expressed in SCZ individuals and revealed a lower frequency compared with the respective miR-137 targets (Additional file 1: Table S6, differentially expressed targets: let-7a 14%, miR-21-5p 11%, miR-93-5p 14%, miR-451a 0%, and miR-675-5p 0%). The numbers of differentially expressed target genes were significantly different between miR-137 and the five pooled controls (*P* = 0.031, *Χ*^2^ test, two-sided, Yates correction, Additional file 1: Table S6).

miR-137 acts cooperatively and synergistically with miR-124 and miR-128 [7, 33, 34]; therefore, changes in the expression of these two microRNAs may interfere with the expression of miR-137 target genes. We analyzed the precursor expression of both microRNAs in the CommonMind data set and found no evidence of differential expression. *MIR124-2HG* (ENSG00000254377) expression was not different between SCZ and control individuals (*P* = 0.8487, expression level 5.948). No data was available for MIR-128 (ENSG00000207654, ENSG00000207625) or for mature microRNAs. To investigate possible co-regulation with miR-137, we looked for additional binding sites in the 3′UTR of differentially expressed miR-137 target genes (Table 1). Several targets listed in Table 1 had putative binding sites for miR-124 (5 out of 16) and miR-128 (1 out of 16) (Additional file 1: Table S7).

Finally, we performed a functional network analysis of validated miR-137 targets (Table 2). Genes involved in *cell death and survival*, *cellular movement*, *gene expression*, and *molecular transport* were enriched. Many target genes were linked to developmental disorders, neurological diseases, and psychological disorders among which SCZ turned out to be the top hit.

**Table 2:** Ingenuity pathway analysis of the 71 previously published experimentally validated miR-137 targets

Category	Diseases and functions	Number of molecules	*P* value
Cell death and survival	Cell death	48	6.43 × 10^−16^
	Apoptosis	46	8.91 × 10^−19^
	Necrosis	41	3.42 × 10^−14^
	Neuronal cell death	17	5.74 × 10^−9^
Cellular movement	Cell movement	39	1.53 × 10^−15^
	Migration of cells	35	7.17 × 10^−14^
Gene expression	Transcription of RNA	34	6.14 × 10^−14^
	Expression of RNA	36	2.90 × 10^−13^
	Transcription of DNA	29	4.30 × 10^−12^
Molecular transport	Transport of molecule	23	4.69 × 10^−7^
	Secretion of molecule	17	1.53 × 10^−10^
	Secretion of neurotransmitter	9	5.53 × 10^−9^
Developmental disorders	Abnormal morphology of embryonic tissue	17	2.36 × 10^−10^
	Growth failure	14	3.30 × 10^−7^
	Dysgenesis	13	5.57 × 10^−7^
Neurological disease	Schizophrenia	14	7.63 × 10^−9^
	Cell death of cortical neurons	9	2.38 × 10^−8^
	Congenital malformation of brain	9	6.18 × 10^−6^
	Brain cancer	8	2.38 × 10^−6^
	Apoptosis of cortical neurons	7	2.75 × 10^−8^
	Neurodegeneration of pyramidal neurons	3	3.94 × 10^−6^
Psychological disorders	Schizophrenia	14	7.63 × 10^−9^
	Disorder of basal ganglia	12	2.10 × 10^−4^
	Mood disorders	9	8.92 × 10^−5^
	Major affective disorder	8	1.97 × 10^−4^
	Depressive disorder	7	1.49 × 10^−4^
	Dyssomnia	4	1.43 × 10^−4^

## Discussion

In this study, we identified *SHANK2* as a novel direct target of miR-137. We discovered that physiological levels of miR-137 regulate *SHANK2* expression, most likely by repressing translation of SHANK2 protein rather than inducing mRNA degradation. Inhibiting effective protein translation is a known mechanism for local fine tuning of gene expression at postsynaptic sites and is in line with previously reported direct miR-137 targets, including Ephrin B2 (*EFNB2)* and the AMPA receptor subunit GluA1 (*GRIA1*) [35, 36]. miR-137 regulates the expression of several proteins that function at glutamatergic synapses, e.g., EFNB2, GluA1, and Mib1 and thereby influences neuronal maturation and signal transduction [8, 35, 36]. AMPA receptors are anchored to the postsynaptic Shank scaffold via PSD-95/Stargazin proteins [37]. SHANK2 and AMPA receptors are both regulated by miR-137 and are important for synaptic maturation and plasticity; therefore, this control by miR-137 may have synergistic effects on synaptic regulation. Beside the regulation of postsynaptic genes, miR-137 has also been shown to regulate presynaptic genes and presynaptic neurotransmitter release [38]. This is supported by the finding that altered miR-137 levels impact synaptic function and neuronal network formation in the mouse hippocampus [36].

Morphological effects caused by altered Shank2 levels in hippocampal neurons do not correlate to the morphological changes found when miR-137 levels are altered. Overexpression of SHANK2 increased spine volume while its downregulation led to reduced spine volume and increased dendritic arborization [39]. In contrast, miR-137 overexpression or inhibition did not affect dendritic spine morphology, whereas miR-137 inhibition only led to a reduced spine density [36]. This difference might be due to a more subtle regulatory effect of miR-137 on Shank2 protein expression compared to an RNAi-mediated knockdown as microRNAs are “fine-tuners” of protein expression. miR-137 regulates the expression of multiple glutamatergic synapse proteins; therefore, subtle regulation of *SHANK2* expression by miR-137 is likely to be physiologically relevant. miR-137 also has subtle effects on the protein expression of its other targets [36]. Different postsynaptic proteins are probably regulated by miR-137 at the same time, which may increase the intensity of the neuronal response.

We showed a regulatory influence of miR-137 on *SHANK2* expression in mouse hippocampal neurons, whereas the relevance in other brain regions and in human neurons warrants future investigation.

miR-137 has been linked to various disorders including ID, ASD, and SCZ [6, 10, 11, 18]. Impaired synaptic plasticity and glutamatergic neurotransmission have been postulated as underlying pathological mechanisms [40]. A heterozygous microdeletion on 1q21.3 encompassing the genes *MIR137* and *DPYD* was previously described in ID and ASD patients [6, 10, 11]. Reduced levels of precursor and mature miR-137 concurrently with significantly increased levels of downstream target genes (*MITF*, *EZH2*, and *KLF4*) were determined in lymphoblastoid cells isolated from two patients with this 1q21.3 microdeletion [6]. Based on our experimental results, we speculate that reduced miR-137 expression may also increase *SHANK2* levels in these patients, which may contribute to the ID and ASD phenotype seen in these patients.

To further investigate the link between miR-137 and SCZ, we analyzed the expression of miR-137 precursor and known miR-137 target genes in postmortem DLPFC samples of SCZ individuals using the CommonMind gene expression data resource [25]. No difference in miR-137 precursor expression was found between SCZ and control individuals. Our study was limited to miR-137 precursor expression analysis as no data of mature miR-137 expression was available. Previous studies have shown no difference in the levels of precursor and mature miR-137 between SCZ and control individuals [31, 41, 42]. miR-137 expression has been investigated in fibroblasts and fibroblast-derived neurons isolated from individuals homozygous for four schizophrenia-associated SNPs at the MIR137 locus. Increased endogenous miR-137 levels were identified in the minor compared to the major allele SNP group in the neurons, but not in fibroblasts [38]. This indicates that regulation of miR-137 expression varies in different cell types. Analyzing miR-137 expression in specific cortical layers, cell types, or even cell compartments might reveal distinct local alterations in the DLPFC of SCZ patients.

Our network analysis revealed that many validated miR-137 target genes are linked to developmental, neurological, and psychiatric disorders, particularly SCZ. Therefore, we analyzed the expression of these genes in the DLPFC, a brain region that is affected in SCZ individuals. Expression of 23% (16/69) of the analyzed target genes was altered in the DLPFC of SCZ compared with control individuals. This change in expression was above the level of target enrichment in a pooled sample of five control microRNAs. This indicated that the miR-137 signaling network might be altered in the DLPFC of SCZ individuals. Five of these differentially expressed target genes have been associated with SCZ *(TCF4*, *SIRT1*, *XIAP*, *GRIA1*, *ZNF804A*) and two with ASD (*RORA*, *KDM5B*), suggesting some overlap between these two disorders. Four miR-137 target genes (*RORA*, *CPLX1*, *TCF4*, *SIRT1*) even show significant differences on the whole-transcriptome level, according to data provided by the CommonMind Consortium [25]. Furthermore, analysis of the CommonMind RNA sequencing data confirmed elevated *GRIA1* mRNA levels in the DLPFC of SCZ individuals [32], indicating impaired synaptic signaling in the DLPFC. The majority of differentially expressed miR-137 target genes (12/16) were only modestly elevated in SCZ individuals, suggesting that small effects of single genes accumulate in the DLPFC, presumably leading to a general impairment of miR-137 signaling. It is important to note that expression of miR-137 target genes is not regulated by miR-137 alone, but by multiple factors, e.g., other microRNAs, epigenetic mechanisms, subcellular localization, synaptic activity, medication, or other environmental factors. We analyzed miR-124 and miR-128, which act cooperatively with miR-137, and obtained no evidence that these two microRNAs influence the differential expression of miR-137 targets. miR-124 precursor expression was not different between SCZ and control individuals and the differentially expressed miR-137 target genes may only slightly be co-regulated by miR-124 and miR-128. Only 23% of the analyzed miR-137 target genes were differentially expressed on the mRNA level between SCZ and control individuals. Most miR-137 targets have been investigated in cancer cell lines and only 13 miR-137 target genes (including *SHANK2*) have been confirmed in neuronal cells; therefore, some of the confirmed targets may not be miR-137 targets in the brain. Expression of *SHANK2* mRNA was not different in the DLPFC of SCZ and control individuals, which may mean that *SHANK2* is not a relevant miR-137 target in the context of SCZ. However, we and others have identified neuronal miR-137 target genes which were regulated on protein [35, 36] and not on mRNA level. Therefore, we conclude that changes in mRNA expression do not reveal the full regulatory potential of miR-137. In this regard, proteomic data from postmortem human brains will be of high value for future SCZ studies.

## Conclusions

We identified a direct regulatory link between microRNA-137 and *SHANK2*, which is of importance for a spectrum of disorders including ID, ASD, and SCZ. Furthermore, evidence was obtained that miR-137 target genes are differentially expressed between healthy controls and SCZ patients offering additional support for the involvement of miR-137 and its target genes in the pathogenesis of neuropsychiatric disorders. Further studies are warranted to address the mutual interaction in conditional knockout mouse models or human-induced pluripotent stem cell-derived neurons.

## Additional file

Additional file 1: **Figure S1.** Images of primary neuronal cultures pre‐ and posttreatment. **Figure S2.** High conservation of the miR‐137 binding site in the SHANK2‐3’UTR and of miR‐137 between different species. **Figure S3.** Relative expression levels of miR‐137 in different human tissues. **Figure S4.** Uncropped Western blot pictures. **Table S1.** ASD risk genes which are predicted or validated miR‐137 targets. **Table S2.** Primers used for cloning, mutagenesis, and screening. Primer sequences are all shown in 5′→3′ orientation. **Table S3.** Origin of total RNA samples used to measure hsa‐miR‐137 relative expression across different tissues (see Additional file 1: Figure S2 for results). **Table S4.** Experimentally validated miR‐137 targets. **Table S5.** Gene expression analysis of 69 validated miR‐137 target genes (including SHANK2) in the CommonMind RNA sequencing data. **Table S6a.** Gene expression analysis of validated targets from five different control microRNAs in the CommonMind RNA sequencing data. Genes labeled in gray withstand correction for multiple testing using the Benjamini-Hochberg method and a FDR of 10%. **Table S6b.** Comparison of the number of differentially expressed target genes of different microRNAs between SCZ and control individuals in the CommonMind RNASeq data. **Table S7.** Analysis of the 3′UTR of the differentially expressed miR-137 genes in the DLPFC between SCZ and control individuals for additional putative miR-124 and miR-128 binding sites. (PDF 1417 kb)

## Abbreviations

ASD: Autism spectrum disorders
BD: Bipolar disorder
DIV: Day in vitro
DLPFC: Dorsolateral prefrontal cortex
DMEM: Dulbecco’s Modified Eagle’s Medium
FBS: Fetal bovine serum
ID: Intellectual disability
mut: miR-137 binding site-mutated 3′UTR
P/S: Penicillin/streptomycin
RT-qPCR: Real-time quantitative PCR
SCZ: Schizophrenia
wt: *SHANK2* 3′UTR wild type sequence
