# Supplementary material for: A direct regulatory link between microRNA-137 and SHANK2: implications for neuropsychiatric disorders
Source: J Neurodev Disord. 2018 Apr 17;10:15. doi: 10.1186/s11689-018-9233-1 (PMC5905159; doi:10.1186/s11689-018-9233-1)
Supplement: Supplementary file 1 — Figure S1. Images of primary neuronal cultures pre‐ and posttreatment. Figure S2. High conservation of the miR‐137 binding site in the SHANK2‐3’UTR and of miR‐137 between different species. Figure S3. Relative expression levels of miR‐137 in different human tissues. Figure S4. Uncropped Western blot pictures. Table S1. ASD risk genes which are predicted or validated miR‐137 targets. Table S2. Primers used for cloning, mutagenesis, and screening. Primer sequences are all shown in 5′→3′ orientation. Table S3. Origin of total RNA samples used to measure hsa‐miR‐137 relative expression across different tissues (see Additional file 1: Figure S2 for results). Table S4. Experimentally validated miR‐137 targets. Table S5. Gene expression analysis of 69 validated miR‐137 target genes (including SHANK2) in the CommonMind RNA sequencing data. Table S6a. Gene expression analysis of validated targets from five different control microRNAs in the CommonMind RNA sequencing data. Genes labeled in gray withstand correction for multiple testing using the Benjamini-Hochberg method and a FDR of 10%. Table S6b. Comparison of the number of differentially expressed target genes of different microRNAs between SCZ and control individuals in the CommonMind RNASeq data. Table S7. Analysis of the 3′UTR of the differentially expressed miR-137 genes in the DLPFC between SCZ and control individuals for additional putative miR-124 and miR-128 binding sites. (PDF 1417 kb) [file 11689_2018_9233_MOESM1_ESM.pdf]

Additional file 1

A direct regulatory link between microRNA-137 and *SHANK2*: Implications for neuropsychiatric disorders

Ana de Sena Cortabitarte[1*], Simone Berkel[1*], Flavia-Bianca Cristian,

Christine Fischer[1], Gudrun A. Rappold[1,2]

*authors contributed equally

1) Institute of Human Genetics, Ruprecht-Karls-University, Heidelberg, Germany

2) Interdisciplinary Center for Neurosciences (IZN), Ruprecht-Karls-University, Heidelberg, Germany

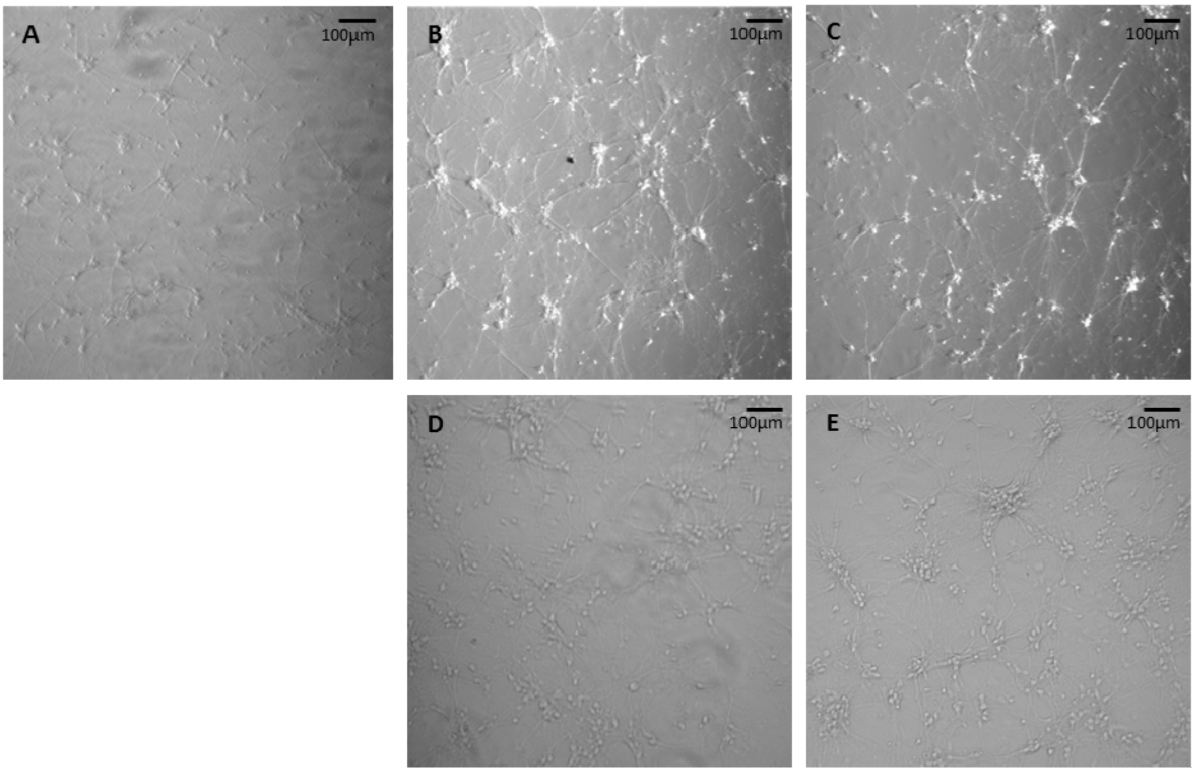

Figure S1. Images of primary neuronal cultures pre- and post-treatment.
(A) Primary hippocampal culture prior to treatment. Post-nucleofection treatment with **(B)** negative control miRNA mimic and **(C)** miR-137 mimic. Cells post-treatment with **(D)** negative control miR-137 inhibitor and **(E)** miR-137 inhibitor.

A

Human	AUCUGCUUAUUUU-GGUACUGA**AAGCAAUA**GUUCCUC
Chimp	AUCUGCUUAUUUU-GGUACUGA**AAGCAAUA**GUUCCUC
Rhesus	AUUUGCUUAUUUU-GGUACUGA**AAGCAAUA**GUUCCUC
Mouse	GACAGUUGAUUUU-GCGC-AGA**AAGCAAUA**GUCCCUC
Rat	GACAGUUGAUUUUUGCAC-AGA**AAGCAAUA**GUCCCUC
Cat	AGUCAAUGGUUUU-GCUCCAGA**AAGCAAUA**GUUCCUC
Dog	AUUCGAUGGUUUU-GCUACAGA**AAGCAAUA**GUUCCUC
Chicken	GUUUGAUGAUUUU-GCUACAGA**AAGCAAUA**GUAACAG
Lizard	GUUUGCUGGUUUU-AUCACAGA**AAGCAAUA**GUAACAG
Xenopus	-------------------------------------

B

hsa-miR-137	U**UAUUGCUU**AAGAAUACGCGUAG
mmu-mir-137	U**UAUUGCUU**AAGAAUACGCGUAG
rno-miR-137	U**UAUUGCUU**AAGAAUACGCGUAG

Figure S 2. High conservation of the miR-137 binding site in the *SHANK2*-3'UTR and of miR-137 between different species.

Sequence alignments were modified from TargetScan Human release 7.1 [http://www.targetscan.org/vert_71/]

(A) The *SHANK2*-3'UTR seed sequence (in bold) for miR-137 is highly conserved among mammals, birds and reptiles (ref. ENSG00000162105.12) **(B)** miR-137 is highly conserved among human (MIMAT0000429), mouse (MIMAT0000149) and rat (MIMAT0000843). The seed sequence is shown in bold.

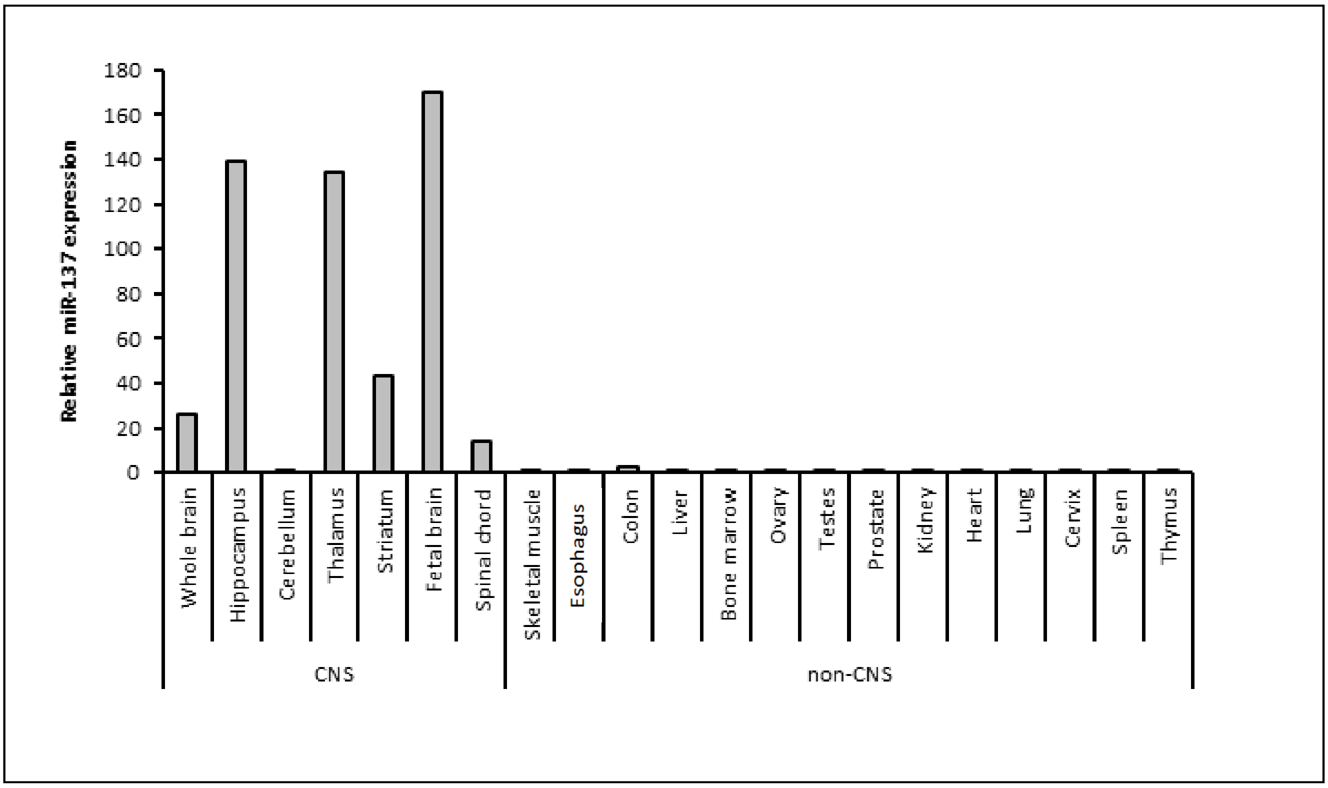

Figure S3. **Relative expression levels of miR-137 in different human tissues.** Relative miR-137 expression was measured with miRCURY LNA ™ PCR primer sets (Exiqon) by RT-qPCR using total RNA samples* from different human tissues.
*see Supplementary Table 3 for sample origin.

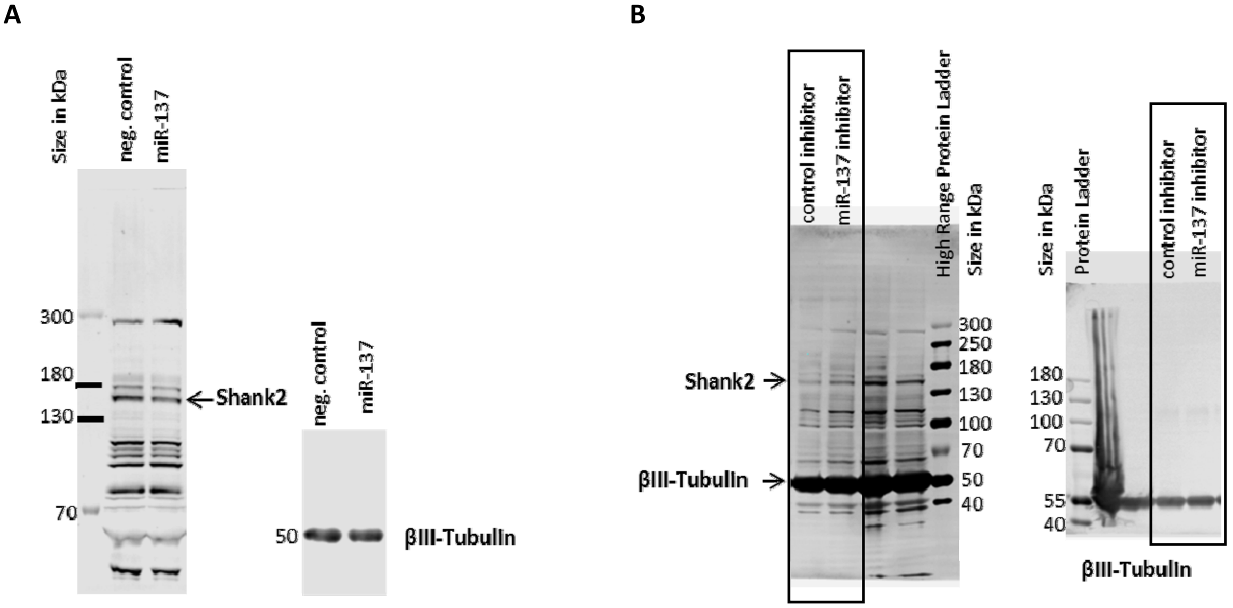

Figure S4. Uncropped western blot pictures. **(A)** Analysis of primary mouse hippocampal neurons 5 days post-transfection with either hsa-miR-137 or control miRNA. **(B)** Analysis of primary mouse hippocampal neurons treated with either hsa-miR-137 or control power inhibitor at DIV5. Protein was harvested after 5 days. The expression of Shank2 and βIII-tubulin could not be quantified on a single membrane, as βIII-tubulin expression was far higher than Shank2expression, resulting in oversaturated βIII-tubulin bands (see B). To overcome this problem we loaded one third of the amount of protein lysate used for Shank2 analysis of the same protein lysates on a second membrane for quantification of the βIII-tubulin bands. The arrow indicates the quantified Shank2 band.

Table S1. ASD risk ge   nes which are predicted or validated miR-137 targets

SFARI Gene ASD candidates	predicted target	validated target
Highh confidence genes		
ADNP		
ANK2		
ARID1B		
ASH1L	+	
ASXL3		
CHD8		
CUL3	+	
DSCAM		
DYRK1A	+	
GRIN2B		
KATNAL2		
KMT5B		
MYTIL		
NAA15	+	
POGZ		
PTEN		
RELN		
SCN2A	+	
SETD5		
SHANK3		
SYNGAP1		
TBR1	+	
TRIP12	+	
Strong candidate genes		
ANKRD11		
BCKDK		
BCL11A		
CACNA1H	+	
CACNA2D3		
CHD2		
CNTN4		
CNTNAP2		
CTNND2		
DEAF1		
ERBIN		
FOXP1	+	
GABRB3		
GIGYF2		
GRIP1	+	
ILF2		
INTS6		
IRF2BPL		
KAT2B	+	
KDM5B	+	
KMT2A	+	
KMT2C		
MAGEL2		
MBOAT7		
MECP2	+	
MED13L		
MET		+
MSNP1AS		
NCKAP1		
NLGN3		
NRXN1	+	
PTCHD1		
RANBP17		
RIMS1		
SCN9A		
SHANK2	+	
SLC6A1	+	
SPAST		
TNRC6B	+	
USP7		
WAC		
WDFY3		

Autism candidate genes from the SFARI (Simons Foundation Autism Research Initiative) Gene database[1, 2] [URL: https://sfari.org/resources/sfari-gene#refs, accessed May 2017] that are predicted targets of miR-137 by TargetScan Human release 7.1 [http://www.targetscan.org/vert_71/] and/or experimentally validated targets of miR-137 (see Supplementary Table 2).

Table S2. Primers used for cloning, mutagenesis and screening. Primer sequences are all shown in 5'→3' orientation.

Cloning Primers

SH2-3UTR_XhoI_F	cagtaattctaggcgatcgcAGAACCTGCACTGACCAACA
SH2-3UTR_XhoI_R	tttaaacgaattcccgggctcgaGGGCATGGAATAGGGCCTC

The XhoI overhang is shown in small letters.

Mutagenesis primers

SH2-3UTR_137mut_F	GCTTATTTTGGTACTGAccGCggTAGTTCCTCATTCAAATCACC
SH2-3UTR_137mut_R	GGTGATTTGAATGAGGAACTAccGCggTCAGTACCAAAATAAGC

The mutated bases are shown in small letters.

Binding site screen primers (m13 tagged)

SH2-3UTRscreen-F-m13	tgtaaaacgacggccagtTGAGGGCCAGATGTTATTCC
SH2-3UTRscreen-R-m13	caggaaacagctatgaccCAAAAGTTGATCTTAAAACCTGGA

The m13 tags are shown in small letters.

RT-qPCR primers - Mouse

Amplicon	Primer name	Sequence 5'-3'
Gapdh	Gapdh-Ex1-3 F	CAGTGCCAGCCTCGTCC
	Gapdh-Ex1-3 R	TGAGGTCAATGAAGGGGTCG
Hprt	Hprt-Ex1-2 F	TCCTCCTCAGACCGCTTTT
	Hprt-Ex1-2 R	CCTGGTTCATCATCGCTAATC
Shank2	Shank2-Ex-5-6 F	TGCTGCCAGTGACTGCATTATTGA
	Shank2-Ex-5-6 R	CAGGGCTGGAAATGCTGGCGT

Table S3. Origin of total RNA samples used to measure hsa-miR-137 relative expression across different tissues (see Supplementary Figure 2 for results).

	Tissue	Name	Origin	Ethnicity	COD/surgery	Company	Catalogue no.	Lot no.
CNS	Whole brain	FirstChoice®Human brain reference RNA	pool (n=23) 23-86 year old males, females	Caucasian		Ambion	AM6050	105P055201A
	Hippocampus	Total RNA human normal hippocampus	27 year old male	Asian	accident	Biochain	R1234052-10	B308094
	Cerebellum	Human brain cerebellum total RNA	pool (n=10) 22-68 year old males, females	Caucasian	sudden death	Clontech	636535	1104145A
		Cerebellum (right)	82 year old male	Caucasian	aortic stenosis	Biochain	R1234041-10	A611366
	Thalamus	Total RNA human normal thalamus	71 year old female	Caucasian	chronic obstructive pulmonary disease	Biochain	R1234079-50	A510113
	Striatum	Adult brain striatum total RNA	20 year old male			Stratagene	530135-41	0460467
	Fetal brain	Total RNA human fetal brain	41 wk gestation male			Biochain	R1244035-50	B308099
	Spinal chord	Total RNA human adult spinal chord	27 year old male	Asian	accident	Biochain	R1234034-50	A804067
non-CNS	Skeletal muscle	Human skeletal muscle total RNA	84 year old female	Caucasian	respiratory arrest	Ambion	AM6000	0803005
			55 year old female	Caucasian	uterine cancer			
			79 year old female	Caucasian	cardiac arrest			
	Esophagus	Human esophagus total RNA	74 year old male	Caucasian	cardiac arrest	Ambion	AM6000	0809006
			75 year old female	Caucasian	cardiac arrest			
			68 year old female	Caucasian	myocardial infarction			
	Colon	Human colon total RNA	23 year old male	Caucasian	blunt force trauma	Ambion	AM6000	0802004
			78 year old female	Caucasian	congestive heart failure			

		75 year old female		congestive heart failure			
Liver	Human liver total RNA	69 year old male		intracranial hemorrhage	Ambion	AM6000	0803004
		64 year old male	Caucasian	intracranial hemorrhage			
		70 year old female	Caucasian	chronic obstructive pulmonary disease			
Bone marrow	Total RNA human normal bone marrow	27 year old male	Asian	accident	Biochain	R1234024-10	B406023
Ovary	Human ovary total RNA	45 year old female	Caucasian	hysterectomy	Ambion	AM6000	0803007
		42 year old female	Caucasian	hysterectomy			
		34 year old female	Caucasian	hysterectomy			
		61 year old female	Caucasian	hysterectomy			
Testes	Human testes total RNA	75 year old male	Caucasian	respiratory arrest	Ambion	AM6000	0803005
		19 year old male	Caucasian	anoxic encephalopathy			
		53 year old male	Caucasian	colon cancer			
Prostate	Human prostate total RNA	79 year old male	Caucasian	Alzheimer's disease	Ambion	AM6000	0805005
		79 year old male	Caucasian	chronic obstructive pulmonary disease			
		72 year old male	Caucasian				
Kidney	Human kidney total RNA	60 year old female	Caucasian	subarachnoid hemorrhage	Ambion	AM6000	0810006
		63 year old female	African American	intracranial hemorrhage			
		62 year old female	Hispanic	intracranial hemorrhage			
Heart	Human heart total RNA	57 year old male	Caucasian	intracranial hemorrhage	Ambion	AM6000	0809006
		77 year old male	Caucasian	cerebral vascular accident			

		71 year old male	Caucasian	congestive heart failure			
		94 year old female	Caucasian	cardiac arrest			
Lung	Human lung total RNA	65 year old male	Caucasian	myocardial infarction	Ambion	AM6000	0809005
		46 year old male	Caucasian	intracranial hemorrhage			
		40 year old female	African American				
Cervix	Human cervix total RNA	42 year old female		hysterectomy	Ambion	AM6000	0809009
		46 year old female	Caucasian	hysterectomy			
		70 year old male	Caucasian	anoxia			
Spleen	Human spleen total RNA	39 year old male	African American	motor vehicle accident	Ambion	AM6000	0810004
		50 year old male	Caucasian	cerebral vascular accident			
		25 year old male	Caucasian	gun shot wound			
Thymus	Human thymus total RNA	6 month old male		surgery	Ambion	AM6000	0812007
		6 month old male		surgery			

Table S4. Experimentally validated miR-137 targets

Gene name	Alternative name	CommonMind/ Ensembl (GRCh38.p7) ID	Present in CommonMind RNAseq dataset	Methods			TargetScan Human 7.1 hit	Original report
				Luciferase reporter assay	RT-qPCR	Western Blot		
AKT2		ENSG00000105221	yes	+		+	yes	[3]
AMPKα1	*PRKAA1*	ENSG00000132356	yes	+	+	+	yes	[4]
AURKA	*Aurora-A*	ENSG00000087586	yes	+		+	no	[5]
BNIP3L	*NIX*	ENSG00000104765	yes	+		+	yes	[6]
C10orf26	*WBP1L*	ENSG00000166272	yes	+			yes	[7]
CACNA1C		ENSG00000151067	yes	+			yes	[7]
CASP3		ENSG00000164305	yes	+		+	no	[8]
CDC42		ENSG00000070831	yes	+		+	yes	[9]
CDK6		ENSG00000105810	yes	+		+	yes	[10]
CENPE		ENSG00000138778	yes	+	+		no	[11]
*CPLX1**		ENSG00000168993	yes	+	+	+	yes	[12]
CSE1L		ENSG00000124207	yes	+	+		yes	[13]
CSMD1		ENSG00000183117	yes	+			yes	[7]
CTBP1		ENSG00000159692	yes	+			yes	[14]
E2F6		ENSG00000169016	yes	-		+	yes	[10]
EFNB2		ENSG00000125266	yes	+	-	+	yes	[15]
ESRRA		ENSG00000173153	yes	+			yes	[16]
*EZH2**		ENSG00000106462	yes	+	-	+	yes	[17]
FMNL2		ENSG00000157827	yes	+	-	+	yes	[18]
FUNDC1		ENSG00000069509	yes	+		+	no	[6]
FXYD6		ENSG00000137726	yes	+	+	+	yes	[19]
GLIPR1	*RTVP-1*	ENSG00000139278	yes	+		+	no	[20]
GRIA1		ENSG00000155511	yes	+	-	+	yes	[21]
*HCRT**		ENSG00000161610	no	+	+		yes	[12]
HEY2		ENSG00000135547	yes	+	+		yes	[22]
HTT		ENSG00000197386	yes	+	+	+	yes	[23]
IBTK		ENSG00000005700	yes	+			yes	[24]
KDM1A	*LSD1*	ENSG00000004487	yes	+	+	+	yes	[25]
KDM5B	*Jarid1B*	ENSG00000117139	yes	+		+	yes	[26]
KLF12		ENSG00000118922	yes	+	-	+	yes	[27]
*KLF4**		ENSG00000136826	yes		+	+	yes	[28, 29]
MET	*c-MET*	ENSG00000105976	yes	+	+	+	no	[30]
*MIB1**		ENSG00000101752	yes	+		+	no	[31]
MITF		ENSG00000187098	yes	+			yes	[32]
MSI1		ENSG00000135097	yes	+		+	yes	[33]
MTDH	*AEG-1*	ENSG00000147649	yes	+		+	yes	[34]
MYO1C		ENSG00000197879	yes	+	-	+	yes	[27]
NCOA2	*TIF2*	ENSG00000140396	yes	-		+	yes	[10]
NEFL		ENSG00000277586	no	+	+	+	no	[35]
NR1I3	*CAR*	ENSG00000143257	yes		+	+	no	[36]
NSF		ENSG00000073969	yes	+	+	+	yes	[12]

NUCKS1		ENSG00000069275	yes	+		+	yes	37
PAQR3		ENSG00000163291	yes	+	+	+	yes	38
*PTBP1**	*LSD1*	ENSG00000011304	yes	+	+	+	yes	39
PTGS2	*Cox-2*	ENSG00000073756	yes	+		+	no	40
PTN		ENSG00000105894	yes		+	+	yes	41
PTP4A3		ENSG00000184489	yes		+	+	yes	42
PXN		ENSG00000089159	yes	+		+	yes	43
RORa		ENSG00000069667	yes	+			yes	44
SERPINA3	*ACT*	ENSG00000196136	yes	+	+		yes	45
SIRT1		ENSG00000096717	yes	+		+	no	26
*SLC6A3**	*DAT*	ENSG00000142319	no	+	+	+	no	46
SMARCA5		ENSG00000153147	yes	+		+	yes	26
SNAI1		ENSG00000124216	no	+	+	+	no	47
SP1		ENSG00000185591	yes	+		+	yes	39
SPTLC1	*SPT*	ENSG00000090054	yes	+		+	yes	48
*STX17**		ENSG00000136874	yes	+	+		yes	12
*SYN3**		ENSG00000185666	yes	+	+	+	yes	12
*SYT1**		ENSG00000067715	yes	+	+	+	yes	12
*SYT7**		ENSG00000011347	yes	+	-		yes	12
TBX3		ENSG00000135111	yes	+	+	+	yes	29
TCF4		ENSG00000196628	yes	+			yes	7
TGFB2		ENSG00000092969	yes	+	+	+	no	35
TRIM13	*CAR*	ENSG00000204977	yes	+	+	+	no	49
TWIST1		ENSG00000122691	yes	+	+	+	yes	3
ULK2		ENSG00000083290	yes	+			yes	24
Wnt7a		ENSG00000154764	yes	+			yes	24
XIAP		ENSG00000101966	yes	+	-	+	no	50
YBX1	*YB1*	ENSG00000065978	yes	+	+	+	yes	30
ZBTB7A	*FBI-1*	ENSG00000178951	yes	+		+	yes	51
ZNF804A		ENSG00000170396	yes	+	+		yes	52

Validated miR-137 targets (n=71) taken from miRTarBase classified with "strong evidence" [http://mirtarbase.mbc.nctu.edu.tw/, accessed January 2017] and/or Pubmed publications of miR-137 targets with experimentally derived evidence from luciferase assay, RT-qPCR and/or Western Blot [https://www.ncbi.nlm.nih.gov/pubmed/, accessed January 2017] (n=71). (-) the respective test was performed but no regulation identified. Most of the targets were investigated in cancer cells, only 12 targets have been validated in neurons, indicated by (*). RNA-Seq data for *HCRT, SLC6A3* and *SNAI1* was not available in the CommonMind data.

Table S5. Gene Expression Analysis of 69 validated miR-137 target genes (including SHANK2) in the CommonMind RNA sequencing data

**Gene-Symbol**	**Ensembl Id**	**Average Expression (log2 CPM)**	**Log2-fold Change**	***P* value**	**FDR estimate 10%**
RORA	ENSG00000069667	7.383	0.118	0.00001	0.00144928
CPLX1	ENSG00000168993	5.455	-0.180	0.00046	0.00289855
TCF4	ENSG00000196628	8.850	0.063	0.00050	0.00434783
SIRT1	ENSG00000096717	5.289	0.074	0.00056	0.0057971
ESRRA	ENSG00000173153	4.619	-0.112	0.00272	0.00724638
CDK6	ENSG00000105810	5.595	0.087	0.00386	0.00869565
XIAP	ENSG00000101966	7.377	0.060	0.00468	0.01014493
ZNF804A	ENSG00000170396	4.131	0.108	0.00529	0.0115942
MET	ENSG00000105976	5.414	0.132	0.00534	0.01304348
CTBP1	ENSG00000159692	6.993	-0.064	0.00577	0.01449275
GRIA1	ENSG00000155511	7.011	0.068	0.00878	0.01594203
KLF12	ENSG00000118922	6.984	0.066	0.01104	0.0173913
MSI1	ENSG00000135097	1.139	-0.135	0.01228	0.01884058
KDM5B	ENSG00000117139	6.007	0.049	0.01454	0.02028986
PAQR3	ENSG00000163291	5.846	0.067	0.01713	0.02173913
TRIM13	ENSG00000204977	5.847	0.050	0.02279	0.02318841
TGFB2	ENSG00000092969	5.517	0.097	0.02685	0.02463768
CDC42	ENSG00000070831	7.534	-0.058	0.03227	0.02608696
KLF4	ENSG00000136826	1.477	-0.172	0.03499	0.02753623
NCOA2	ENSG00000140396	6.747	0.047	0.03542	0.02898551
EZH2	ENSG00000106462	1.354	0.073	0.04909	0.03043478
SYN3	ENSG00000185666	4.613	-0.084	0.05595	0.03188406
PTP4A3	ENSG00000184489	2.837	-0.091	0.05676	0.03333333
SP1	ENSG00000185591	5.599	0.047	0.08631	0.03478261
FUNDC1	ENSG00000069509	4.656	-0.038	0.09195	0.03623188
CENPE	ENSG00000138778	2.748	0.069	0.09479	0.03768116
SYT1	ENSG00000067715	10.210	0.065	0.11090	0.03913043
STX17	ENSG00000136874	4.629	0.027	0.12410	0.04057971
EFNB2	ENSG00000125266	5.609	0.033	0.12640	0.04202899
CASP3	ENSG00000164305	4.559	0.036	0.12750	0.04347826
NR1I3	ENSG00000143257	1.479	0.049	0.13600	0.04492754
ZBTB7A	ENSG00000178951	5.027	-0.068	0.14460	0.04637681
TWIST1	ENSG00000122691	0.638	-0.084	0.14960	0.04782609
WNT7A	ENSG00000154764	3.108	-0.065	0.16770	0.04927536
MIB1	ENSG00000101752	8.175	0.025	0.18000	0.05072464
MITF	ENSG00000187098	3.410	0.039	0.21220	0.05217391
FMNL2	ENSG00000157827	8.187	0.035	0.22180	0.05362319
YBX1	ENSG00000065978	5.940	-0.041	0.29220	0.05507246
SPTLC1	ENSG00000090054	5.388	-0.023	0.29430	0.05652174
GLIPR1	ENSG00000139278	5.016	0.036	0.30640	0.05797101
CSMD1	ENSG00000183117	7.335	0.032	0.33240	0.05942029
SERPINA3	ENSG00000196136	4.049	0.191	0.33810	0.06086957
HTT	ENSG00000197386	7.895	0.027	0.34100	0.06231884
BNIP3L	ENSG00000104765	8.152	0.024	0.35000	0.06376812
NUCKS1	ENSG00000069275	9.121	0.021	0.35110	0.06521739
SHANK2	ENSG00000162105	6.152	0.039	0.37700	0.06666667
AURKA	ENSG00000087586	1.580	-0.027	0.38980	0.06811594
PTN	ENSG00000105894	6.949	-0.032	0.39450	0.06956522
PTBP1	ENSG00000011304	4.071	-0.029	0.41500	0.07101449
IBTK	ENSG00000005700	7.166	0.016	0.41580	0.07246377
HEY2	ENSG00000135547	1.822	-0.035	0.43150	0.07391304
MTDH	ENSG00000147649	7.392	0.013	0.46170	0.07536232
CACNA1C	ENSG00000151067	5.727	-0.025	0.46280	0.07681159
PXN	ENSG00000089159	2.618	0.033	0.47190	0.07826087
NSF	ENSG00000073969	8.272	0.026	0.50060	0.07971014
SYT7	ENSG00000011347	6.592	-0.027	0.50140	0.08115942
PTGS2	ENSG00000073756	3.938	0.036	0.61690	0.0826087
CSE1L	ENSG00000124207	6.821	0.010	0.62230	0.08405797
PRKAA1	ENSG00000132356	6.339	-0.012	0.62540	0.08550725
TBX3	ENSG00000135111	1.470	-0.028	0.65350	0.08695652
SMARCA5	ENSG00000153147	8.061	0.009	0.65700	0.0884058
KDM1A	ENSG00000004487	6.423	-0.006	0.72460	0.08985507
FXYD6	ENSG00000137726	6.021	0.013	0.72830	0.09130435
ULK2	ENSG00000083290	6.758	-0.005	0.75000	0.09275362
NEFL	ENSG00000104725	9.387	-0.009	0.79920	0.0942029
WBP1L	ENSG00000166272	4.769	-0.006	0.79940	0.09565217
MYO1C	ENSG00000197879	3.140	0.007	0.85080	0.09710145
AKT2	ENSG00000105221	5.312	0.002	0.94750	0.09855072
E2F6	ENSG00000169016	4.401	-0.001	0.95580	0.1

Genes labeled in grey withstand the correction for multiple testing using the Benjamini Hochberg Method and a FDR of 10%. Log2 CPM - log2 counts per million.

Table S6a. Gene expression analysis of validated targets from 5 different control microRNAs in the CommonMind RNA sequencing data. Genes labeled in grey withstand correction for multiple testing using the Benjamini Hochberg Method and a FDR of 10%.

let7a-5p		
Gene-Symbol	P-value	FDR estimate 10%
IGF2	0.00001149	0.0020
E2F1	0.0006563	0.0041
RAB40C	0.0009859	0.0061
CDK6	0.00386	0.0082
CDC34	0.006466	0.0102
KRAS	0.007222	0.0122
ARG2	0.01264	0.0143
TNFRSF10B	0.01835	0.0163
BCL2	0.02829	0.0184
UHRF2	0.03827	0.0204
TUSC2	0.04392	0.0224
EZH2	0.04909	0.0245
STAT3	0.05656	0.0265
HRAS	0.05856	0.0286
DICER1	0.06955	0.0306
LIN28B	0.07554	0.0327
ITGB3	0.07761	0.0347
MAP4K4	0.07967	0.0367
ZFP36L1	0.08988	0.0388
TMED7	0.09176	0.0408
NFKB1	0.1111	0.0429
NKIRAS2	0.1213	0.0449
CASP3	0.1275	0.0469
THBS1	0.1288	0.0490
HMGA1	0.1391	0.0510
HAS2	0.1492	0.0531
CCND2	0.1868	0.0551
AMMECR1	0.1988	0.0571
NF2	0.2049	0.0592
TGFBR3	0.2088	0.0612
CDKN1A	0.2387	0.0633
TNFAIP3	0.2659	0.0653
USP35	0.3224	0.0673
RAVER2	0.4171	0.0694
EGFR	0.4233	0.0714
MYC	0.4492	0.0735
PARP1	0.4517	0.0755
SLC20A1	0.4853	0.0776
EWSR1	0.4863	0.0796
CASP8	0.4963	0.0816
PAK1	0.5672	0.0837
EGR3	0.6876	0.0857
PKM	0.6922	0.0878
CASP9	0.7121	0.0898
NRAS	0.766	0.0918
NEFM	0.796	0.0939
MEIS1	0.8293	0.0959
PRDM1	0.8572	0.0980
APP	0.9402	0.1000
TRIM71	nd	
FOXA1	nd	
NR1I2	nd	
VDR	nd	
HMGA2	nd	
AGO4	nd	
LIN28A	nd	
HNRNPDL	nd	
IL6	nd	
E2F2	nd	
IGF2BP1	nd	
MPL	nd	

miR-93-5p		
Gene-Symbol	P-value	FDR estimate 10%
RPS6KA4	0.0001	0.0023
E2F1	0.0007	0.0045
RAB11FIP1	0.0017	0.0068
MXD1	0.0017	0.0091
PHLPP2	0.0037	0.0114
MGLL	0.0132	0.0136
VEGFA	0.0162	0.0159
SNX16	0.0283	0.0182
PDCD4	0.0323	0.0205
STK11	0.0439	0.0227
TUSC2	0.0439	0.0250
NPAS2	0.0460	0.0273
PTEN	0.0585	0.0295
PURA	0.0695	0.0318
PTENP1	0.1039	0.0341
WNT2B	0.1150	0.0364
ICAM1	0.1162	0.0386
FOXO3	0.1395	0.0409
RHOC	0.1652	0.0432
SASH1	0.1669	0.0455
HIF1A	0.2201	0.0477
CDKN1A	0.2387	0.0500
ATG16L1	0.2686	0.0523
LATS2	0.3053	0.0545
SMAD7	0.3353	0.0568
MYC	0.4492	0.0591
HLA-F	0.4499	0.0614
TGFBR2	0.4531	0.0636
TRIP10	0.5000	0.0659
TGFB1	0.5342	0.0682
SLC16A9	0.6328	0.0705
ZNRF3	0.6426	0.0727
TP53INP1	0.6735	0.0750
SLC2A4	0.6935	0.0773
ZBTB4	0.7260	0.0795
CERS2	0.7356	0.0818
ITGB8	0.7435	0.0841
SNX9	0.7488	0.0864
MSMO1	0.7712	0.0886
KAT2B	0.7981	0.0909
MAPK9	0.8063	0.0932
ABCA1	0.8599	0.0955
NFKBIA	0.9764	0.0977
DAB2	0.9883	0.1000
EREG	nd	
CXCL8	nd	
ANG	nd	
MMP3	nd	
FOXA1	nd	

CCR7	nd
RRM2	nd
AGO1	nd
AURKB	nd
WNT1	nd

miR-21-5p		
Gene-Symbol	P-value	FDR estimate 10%
E2F1	0.0000007	0.0008
DERL1	0.0000369	0.0015
APAF1	0.0003	0.0023
BCL6	0.0004	0.0030
RMND5A	0.0008	0.0038
JMY	0.0017	0.0045
RASA1	0.0020	0.0053
TIAM1	0.0024	0.0060
PELI1	0.0025	0.0068
RHOB	0.0033	0.0075
YOD1	0.0066	0.0083
WWP1	0.0071	0.0090
BMPR2	0.0076	0.0098
CADM1	0.0092	0.0105
STUB1	0.0101	0.0113
IRAK1	0.0139	0.0120
VEGFA	0.0162	0.0128
SOX5	0.0169	0.0135
FBXO11	0.0175	0.0143
TNFRSF10B	0.0184	0.0150
LRP6	0.0195	0.0158
TOR1AIP2	0.0196	0.0165
SOD3	0.0226	0.0173
SIRT2	0.0238	0.0180
NCOA3	0.0243	0.0188
MSH6	0.0263	0.0195
TGFB2	0.0269	0.0203
BCL2	0.0283	0.0211
MEF2C	0.0308	0.0218
PDCD4	0.0323	0.0226
ANP32A	0.0326	0.0233
SETD2	0.0363	0.0241
PLAT	0.0404	0.0248
SMN1	0.0404	0.0256
LRRFIP1	0.0517	0.0263
PTPN14	0.0542	0.0271
STAT3	0.0566	0.0278
PLOD3	0.0573	0.0286
PTEN	0.0585	0.0293
TGIF1	0.0719	0.0301
SP1	0.0863	0.0308
DOCK7	0.0869	0.0316
FZD6	0.0872	0.0323
NFIB	0.0905	0.0331
PIK3R1	0.0955	0.0338
TAP1	0.1003	0.0346
BMI1	0.1024	0.0353
VHL	0.1049	0.0361
JAG1	0.1056	0.0368
NFKB1	0.1111	0.0376
SECISBP2L	0.1141	0.0383
SATB1	0.1142	0.0391
ICAM1	0.1162	0.0398
CDK2AP1	0.1214	0.0406
CBX4	0.1254	0.0414
ISCU	0.1303	0.0421
ZBTB7A	0.1446	0.0429
PSMD9	0.1579	0.0436

miR-451a		
Gene-Symbol	P-value	FDR estimate 10%
FRZB	0.005963	0.0043
RAB14	0.01565	0.0087
AKT1	0.01737	0.0130
ADAM10	0.028	0.0174
BCL2	0.02829	0.0217
PKD1	0.03512	0.0261
DCBLD2	0.04559	0.0304
MIF	0.06391	0.0348
TMED7	0.09176	0.0391
IL6R	0.1152	0.0435
TSC1	0.1531	0.0478
CPNE3	0.1699	0.0522
CDKN2D	0.1942	0.0565
CAB39	0.2041	0.0609
IKBKB	0.2547	0.0652
MMP2	0.3077	0.0696
RAB5A	0.3113	0.0739
OXTR	0.4005	0.0783
MYC	0.4492	0.0826
MAP3K1	0.4603	0.0870
MAPK1	0.5261	0.0913
ROR2	0.7696	0.0957
ABCB1	0.8884	0.1000
MMP9	nd	
OSR1	nd	
IL6	nd	

miR-675-5p		
Gene-Symbol	P-value	FDR estimate 10%
RB1	0.03681	0.0067
CALN1	0.1022	0.0133
GPR55	0.1959	0.0200
CDC6	0.1997	0.0267
MITF	0.2122	0.0333
REPS2	0.2144	0.0400
ATP8A2	0.2295	0.0467
HDAC5	0.3324	0.0533
DDB2	0.4671	0.0600
NOMO1	0.4899	0.0667
TGFB1	0.5342	0.0733
TGFBI	0.5424	0.0800
RUNX1	0.5603	0.0867
HDAC4	0.6904	0.0933
HDAC6	0.9235	0.1000

RECK	0.1601	0.0444
HPGD	0.1660	0.0451
SASH1	0.1669	0.0459
DDAH1	0.1685	0.0466
MARCKS	0.1704	0.0474
IL12A	0.1718	0.0481
IGF1R	0.1721	0.0489
ELAVL4	0.1974	0.0496
FMOD	0.2065	0.0504
MAP2K3	0.2087	0.0511
TGFBR3	0.2088	0.0519
TRAF7	0.2166	0.0526
REST	0.2175	0.0534
HNRNPK	0.2510	0.0541
TM9SF3	0.2543	0.0549
SOCS6	0.2602	0.0556
TNFAIP3	0.2659	0.0564
TOPORS	0.2697	0.0571
TIMP3	0.2794	0.0579
HMGB1	0.2924	0.0586
MMP2	0.3077	0.0594
NFIA	0.3089	0.0602
SPRY2	0.3120	0.0609
TPM1	0.3204	0.0617
SMAD7	0.3353	0.0624
DOCK4	0.3457	0.0632
CLU	0.3563	0.0639
RPS7	0.3607	0.0647
ERBB2	0.3739	0.0654
OXTR	0.4005	0.0662
BASP1	0.4217	0.0669
EGFR	0.4233	0.0677
TP53BP2	0.4256	0.0684
MYC	0.4492	0.0692
TGFBR2	0.4531	0.0699
COL4A1	0.4532	0.0707
CASP8	0.4963	0.0714
RASGRP1	0.5025	0.0722
SOX2	0.5228	0.0729
PPIF	0.5240	0.0737
DNM1L	0.5280	0.0744
ICOSLG	0.5327	0.0752
TGFB1	0.5342	0.0759
TGFBI	0.5424	0.0767
DUSP10	0.5557	0.0774
PCGF2	0.5632	0.0782
TLR3	0.5763	0.0789
EGLN1	0.6367	0.0797
HIPK3	0.6425	0.0805
FOXO1	0.6607	0.0812
GAS5	0.6694	0.0820
MSH2	0.6728	0.0827
SERPINI1	0.6760	0.0835
MTAP	0.6775	0.0842
EIF4A2	0.6882	0.0850
ANKRD46	0.6945	0.0857
UBE2N	0.6961	0.0865
DAXX	0.7191	0.0872
BTG2	0.7284	0.0880
DOCK5	0.7577	0.0887
CDC25A	0.7601	0.0895
PCBP1	0.7763	0.0902
SMARCA4	0.7771	0.0910
PPARA	0.7837	0.0917
CEBPB	0.8096	0.0925
RFFL	0.8193	0.0932
NAV3	0.8407	0.0940
PIAS3	0.8593	0.0947
RTN4	0.8882	0.0955
ABCB1	0.8884	0.0962
MYD88	0.8896	0.0970
CCR1	0.9415	0.0977

AKT2	0.9475	0.0985
BCL10	0.9579	0.0992
CASC2	0.9643	0.1000
NCAPG	nd	
E2F2	nd	
SERPINB5	nd	
TP63	nd	
IL1B	nd	
PTX3	nd	
CCL20	nd	
NTF3	nd	
FASLG	nd	
TCF21	nd	
MMP9	nd	
GDF5	nd	
CXCL10	nd	
RHO	nd	
TICAM2	nd	
COX2	nd	
FOXP3	nd	
MSLN	nd	
SOCS1	nd	

nd – no data available

Table S6b. Comparison of the number of differentially expressed target genes of different microRNAs between SCZ and control individuals in the CommonMind RNASeq data

microRNA	Amount of differentially expressed targets (%)	Differentially vs. non-differentially expressed targets
**let-7a**	14	7/42
**miR-21-5p**	11	15/118
**miR-93-5p**	14	6/38
**miR-451a**	0	0/23
**miR-675-5p**	0	0/15
**miR-137**	23	16/53

	Differentially expressed target genes	Non-differentially expressed target genes
**Total controls (pooled 5 controls)**	28	236
**Controls after correction for gene overlaps (counting each gene once)**	27	209
**Controls after correction for target gene overlaps with miR-137**	27	201
**miR-137 targets after correction for gene overlaps with controls**	16	45

The corrected data labeled in grey was used to perform a frequency comparison using a two-sided X^2-test with Yates correction that revealed a *P*-value of 0.009.

Table S7. Analysis of the 3´UTR of the differentially expressed miR-137 genes in the DLPFC between SCZ and control individuals for additional putative miR-124 and miR-128 binding sites.

Gene Symbol	miR-124	miR-128
RORA	+	
CPLX1		
TCF4	+	+
SIRT1	+	
ESRRA		
CDK6		
XIAP		
ZNF804A		
MET	+	
CTBP1		
GRIA1	+	
KLF12		
MSI1		
KDM5B		
PAQR3		
TRIM13		

References

1. Abrahams BS, Arking DE, Campbell DB, Mefford HC, Morrow EM, Weiss LA *et al.* SFARI Gene 2.0: a community-driven knowledgebase for the autism spectrum disorders (ASDs). *Molecular autism* 2013; **4**(1)**:** 36.

2. Banerjee-Basu S, Packer A. SFARI Gene: an evolving database for the autism research community. *Disease models & mechanisms* 2010; **3**(3-4)**:** 133-135.

3. Liu LL, Lu SX, Li M, Li LZ, Fu J, Hu W *et al.* FoxD3-regulated microRNA-137 suppresses tumour growth and metastasis in human hepatocellular carcinoma by targeting AKT2. *Oncotarget* 2014; **5**(13)**:** 5113-5124.

4. Sollis E, Graham SA, Vino A, Froehlich H, Vreeburg M, Dimitropoulou D *et al.* Identification and functional characterization of de novo FOXP1 variants provides novel insights into the etiology of neurodevelopmental disorder. *Hum Mol Genet* 2016; **25**(3)**:** 546-557.

5. Chang X, Zhang H, Lian S, Zhu W. miR-137 suppresses tumor growth of malignant melanoma by targeting aurora kinase A. *Biochem Biophys Res Commun* 2016; **475**(3)**:** 251-256.

6. Li W, Zhang X, Zhuang H, Chen HG, Chen Y, Tian W *et al.* MicroRNA-137 is a novel hypoxia-responsive microRNA that inhibits mitophagy via regulation of two mitophagy receptors FUNDC1 and NIX. *J Biol Chem* 2014; **289**(15)**:** 10691-10701.

7. Kwon E, Wang W, Tsai LH. Validation of schizophrenia-associated genes CSMD1, C10orf26, CACNA1C and TCF4 as miR-137 targets. *Mol Psychiatry* 2013; **18**(1)**:** 11-12.

8. Su TJ, Ku WH, Chen HY, Hsu YC, Hong QS, Chang GC *et al.* Oncogenic miR-137 contributes to cisplatin resistance via repressing CASP3 in lung adenocarcinoma. *Am J Cancer Res* 2016; **6**(6)**:** 1317-1330.

9. Liu M, Lang N, Qiu M, Xu F, Li Q, Tang Q *et al.* miR-137 targets Cdc42 expression, induces cell cycle G1 arrest and inhibits invasion in colorectal cancer cells. *Int J Cancer* 2011; **128**(6)**:** 1269-1279.

10. Kozaki K, Imoto I, Mogi S, Omura K, Inazawa J. Exploration of tumor-suppressive microRNAs silenced by DNA hypermethylation in oral cancer. *Cancer Res* 2008; **68**(7)**:** 2094-2105.

11. Liang ML, Hsieh TH, Ng KH, Tsai YN, Tsai CF, Chao ME *et al.* Downregulation of miR-137 and miR-6500-3p promotes cell proliferation in pediatric high-grade gliomas. *Oncotarget* 2016; **7**(15)**:** 19723-19737.

12. Siegert S, Seo J, Kwon EJ, Rudenko A, Cho S, Wang W *et al.* The schizophrenia risk gene product miR-137 alters presynaptic plasticity. *Nat Neurosci* 2015; **18**(7)**:** 1008-1016.

13. Li KK, Yang L, Pang JC, Chan AK, Zhou L, Mao Y *et al.* MIR-137 suppresses growth and invasion, is downregulated in oligodendroglial tumors and targets CSE1L. *Brain Pathol* 2013; **23**(4)**:** 426-439.

14. Deng Y, Deng H, Bi F, Liu J, Bemis LT, Norris D *et al.* MicroRNA-137 targets carboxyl-terminal binding protein 1 in melanoma cell lines. *Int J Biol Sci* 2011; **7**(1)**:** 133-137.

15. Wu S, Zhang R, Nie F, Wang X, Jiang C, Liu M *et al.* MicroRNA-137 Inhibits EFNB2 Expression Affected by a Genetic Variant and Is Expressed Aberrantly in Peripheral Blood of Schizophrenia Patients. *EBioMedicine* 2016; **12:** 133-142.

16. Zhao Y, Li Y, Lou G, Zhao L, Xu Z, Zhang Y *et al.* MiR-137 targets estrogen-related receptor alpha and impairs the proliferative and migratory capacity of breast cancer cells. *PLoS One* 2012; **7**(6)**:** e39102.

17. Szulwach KE, Li X, Smrt RD, Li Y, Luo Y, Lin L *et al.* Cross talk between microRNA and epigenetic regulation in adult neurogenesis. *J Cell Biol* 2010; **189**(1)**:** 127-141.

18. Liang L, Li X, Zhang X, Lv Z, He G, Zhao W *et al.* MicroRNA-137, an HMGA1 target, suppresses colorectal cancer cell invasion and metastasis in mice by directly targeting FMNL2. *Gastroenterology* 2013; **144**(3)**:** 624-635 e624.

19. Li ZM, Zhang HY, Wang YX, Wang WB. MicroRNA-137 is downregulated in human osteosarcoma and regulates cell proliferation and migration through targeting FXYD6. *J Drug Target* 2016; **24**(2)**:** 102-110.

20. Bier A, Giladi N, Kronfeld N, Lee HK, Cazacu S, Finniss S *et al.* MicroRNA-137 is downregulated in glioblastoma and inhibits the stemness of glioma stem cells by targeting RTVP-1. *Oncotarget* 2013; **4**(5)**:** 665-676.

21. Olde Loohuis NF, Ba W, Stoerchel PH, Kos A, Jager A, Schratt G *et al.* MicroRNA-137 Controls AMPA-Receptor-Mediated Transmission and mGluR-Dependent LTD. *Cell Rep* 2015; **11**(12)**:** 1876-1884.

22. Wu DC, Zhang MF, Su SG, Fang HY, Wang XH, He D *et al.* HEY2, a target of miR-137, indicates poor outcomes and promotes cell proliferation and migration in hepatocellular carcinoma. *Oncotarget* 2016; **7**(25)**:** 38052-38063.

23. Kozlowska E, Krzyzosiak WJ, Koscianska E. Regulation of huntingtin gene expression by miRNA-137, -214, -148a, and their respective isomiRs. *Int J Mol Sci* 2013; **14**(8)**:** 16999-17016.

24. Zhang B, Ma Z, Li X, Zhang C, Shao Y, Liu Z *et al.* Tanshinones suppress non-small cell lung cancer through up-regulating miR-137. *Acta Biochim Biophys Sin (Shanghai)* 2016; **48**(8)**:** 768-770.

25. Balaguer F, Link A, Lozano JJ, Cuatrecasas M, Nagasaka T, Boland CR *et al.* Epigenetic silencing of miR-137 is an early event in colorectal carcinogenesis. *Cancer Res* 2010; **70**(16)**:** 6609-6618.

26. Tarantino C, Paolella G, Cozzuto L, Minopoli G, Pastore L, Parisi S *et al.* miRNA 34a, 100, and 137 modulate differentiation of mouse embryonic stem cells. *FASEB J* 2010; **24**(9)**:** 3255-3263.

27. Du Y, Chen Y, Wang F, Gu L. miR-137 plays tumor suppressor roles in gastric cancer cell lines by targeting KLF12 and MYO1C. *Tumour Biol* 2016; **37**(10)**:** 13557-13569.

28. Willemsen MH, Valles A, Kirkels LA, Mastebroek M, Olde Loohuis N, Kos A *et al.* Chromosome 1p21.3 microdeletions comprising DPYD and MIR137 are associated with intellectual disability. *Journal of medical genetics* 2011; **48**(12)**:** 810-818.

29. Jiang K, Ren C, Nair VD. MicroRNA-137 represses Klf4 and Tbx3 during differentiation of mouse embryonic stem cells. *Stem Cell Res* 2013; **11**(3)**:** 1299-1313.

30. Luo C, Tetteh PW, Merz PR, Dickes E, Abukiwan A, Hotz-Wagenblatt A *et al.* miR-137 inhibits the invasion of melanoma cells through downregulation of multiple oncogenic target genes. *J Invest Dermatol* 2013; **133**(3)**:** 768-775.

31. Smrt RD, Szulwach KE, Pfeiffer RL, Li X, Guo W, Pathania M *et al.* MicroRNA miR-137 regulates neuronal maturation by targeting ubiquitin ligase mind bomb-1. *Stem cells* 2010; **28**(6)**:** 1060-1070.

32. Haflidadottir BS, Bergsteinsdottir K, Praetorius C, Steingrimsson E. miR-148 regulates Mitf in melanoma cells. *PLoS One* 2010; **5**(7)**:** e11574.

33. Smith AR, Marquez RT, Tsao WC, Pathak S, Roy A, Ping J *et al.* Tumor suppressive microRNA-137 negatively regulates Musashi-1 and colorectal cancer progression. *Oncotarget* 2015; **6**(14)**:** 12558-12573.

34. Guo J, Xia B, Meng F, Lou G. miR-137 suppresses cell growth in ovarian cancer by targeting AEG-1. *Biochem Biophys Res Commun* 2013; **441**(2)**:** 357-363.

35. Tamim S, Vo DT, Uren PJ, Qiao M, Bindewald E, Kasprzak WK *et al.* Genomic analyses reveal broad impact of miR-137 on genes associated with malignant transformation and neuronal differentiation in glioblastoma cells. *PLoS One* 2014; **9**(1)**:** e85591.

36. Chen S, He N, Yu J, Li L, Hu Y, Deng R *et al.* Post-transcriptional regulation by miR-137 underlies the low abundance of CAR and low rate of bilirubin clearance in neonatal mice. *Life Sci* 2014; **107**(1-2)**:** 8-13.

37. Shen H, Wang L, Ge X, Jiang CF, Shi ZM, Li DM *et al.* MicroRNA-137 inhibits tumor growth and sensitizes chemosensitivity to paclitaxel and cisplatin in lung cancer. *Oncotarget* 2016; **7**(15)**:** 20728-20742.

38. Xiu Y, Liu Z, Xia S, Jin C, Yin H, Zhao W *et al.* MicroRNA-137 upregulation increases bladder cancer cell proliferation and invasion by targeting PAQR3. *PLoS One* 2014; **9**(10)**:** e109734.

39. Sun G, Ye P, Murai K, Lang MF, Li S, Zhang H *et al.* miR-137 forms a regulatory loop with nuclear receptor TLX and LSD1 in neural stem cells. *Nat Commun* 2011; **2:** 529.

40. Chen L, Wang X, Wang H, Li Y, Yan W, Han L *et al.* miR-137 is frequently down-regulated in glioblastoma and is a negative regulator of Cox-2. *Eur J Cancer* 2012; **48**(16)**:** 3104-3111.

41. Xiao J, Peng F, Yu C, Wang M, Li X, Li Z *et al.* microRNA-137 modulates pancreatic cancer cells tumor growth, invasion and sensitivity to chemotherapy. *Int J Clin Exp Pathol* 2014; **7**(11)**:** 7442-7450.

42. Wang L, Liu J, Zhong Z, Gong X, Liu W, Shi L *et al.* PTP4A3 is a target for inhibition of cell proliferatin, migration and invasion through Akt/mTOR signaling pathway in glioblastoma under the regulation of miR-137. *Brain Res* 2016; **1646:** 441-450.

43. Bi Y, Han Y, Bi H, Gao F, Wang X. miR-137 impairs the proliferative and migratory capacity of human non-small cell lung cancer cells by targeting paxillin. *Hum Cell* 2014; **27**(3)**:** 95-102.

44. Devanna P, Vernes SC. A direct molecular link between the autism candidate gene RORa and the schizophrenia candidate MIR137. *Scientific reports* 2014; **4:** 3994.

45. Lok SI, van Mil A, Bovenschen N, van der Weide P, van Kuik J, van Wichen D *et al.* Post-transcriptional regulation of alpha-1-antichymotrypsin by microRNA-137 in chronic heart failure and mechanical support. *Circ Heart Fail* 2013; **6**(4)**:** 853-861.

46. Jia X, Wang F, Han Y, Geng X, Li M, Shi Y *et al.* miR-137 and miR-491 Negatively Regulate Dopamine Transporter Expression and Function in Neural Cells. *Neurosci Bull* 2016; **32**(6)**:** 512-522.

47. Dong P, Xiong Y, Watari H, Hanley SJ, Konno Y, Ihira K *et al.* MiR-137 and miR-34a directly target Snail and inhibit EMT, invasion and sphere-forming ability of ovarian cancer cells. *J Exp Clin Cancer Res* 2016; **35**(1)**:** 132.

48. Geekiyanage H, Chan C. MicroRNA-137/181c regulates serine palmitoyltransferase and in turn amyloid beta, novel targets in sporadic Alzheimer's disease. *J Neurosci* 2011; **31**(41)**:** 14820-14830.

49. Takwi AA, Wang YM, Wu J, Michaelis M, Cinatl J, Chen T. miR-137 regulates the constitutive androstane receptor and modulates doxorubicin sensitivity in parental and doxorubicin-resistant neuroblastoma cells. *Oncogene* 2014; **33**(28)**:** 3717-3729.

50. Li X, Chen W, Zeng W, Wan C, Duan S, Jiang S. microRNA-137 promotes apoptosis in ovarian cancer cells via the regulation of XIAP. *Br J Cancer* 2017; **116**(1)**:** 66-76.

51. Zhu M, Li M, Wang T, Linghu E, Wu B. MicroRNA-137 represses FBI-1 to inhibit proliferation and in vitro invasion and migration of hepatocellular carcinoma cells. *Tumour Biol* 2016; **37**(10)**:** 13995-14008.

52. Kim AH, Parker EK, Williamson V, McMichael GO, Fanous AH, Vladimirov VI. Experimental validation of candidate schizophrenia gene ZNF804A as target for hsa-miR-137. *Schizophr Res* 2012; **141**(1)**:** 60-64.
